# Towards an Electrochemical Immunosensor System with Temperature Control for Cytokine Detection

**DOI:** 10.3390/s18051309

**Published:** 2018-04-24

**Authors:** Julia Metzner, Katrin Luckert, Karin Lemuth, Martin Hämmerle, Ralf Moos

**Affiliations:** 1 Robert Bosch GmbH, Corporate Research, Robert-Bosch-Campus 1, D-71272 Renningen, Germany; 2 Department of Functional Materials, University of Bayreuth, Universitätsstraße 30, D-95440 Bayreuth, Germany; functional.materials@uni-bayreuth.de

**Keywords:** cytokines, electrochemical immunosensor, fluidic system, temperature control

## Abstract

The cytokine interleukin-13 (IL-13) plays a major role in airway inflammation and is a target of new anti-asthmatic drugs. Hence, IL-13 determination could be interesting in assessing therapy success. Thus, in this work an electrochemical immunosensor for IL-13 was developed and integrated into a fluidic system with temperature control for read-out. Therefore, two sets of results are presented. First, the sensor was set up in sandwich format on single-walled carbon nanotube electrodes and was read out by applying the hydrogen peroxide–hydroquinone–horseradish peroxidase (HRP) system. Second, a fluidic system was built up with an integrated heating function realized by Peltier elements that allowed a temperature-controlled read-out of the immunosensor in order to study the influence of temperature on the amperometric read-out. The sensor was characterized at the temperature optimum of HRP at 30 °C and at 12 °C as a reference for lower performance. These results were compared to a measurement without temperature control. At the optimum operation temperature of 30 °C, the highest sensitivity (slope) was obtained compared to lower temperatures and a limit of detection of 5.4 ng/mL of IL-13 was calculated. Taken together, this approach is a first step towards an automated electrochemical immunosensor platform and shows the potential of a temperature-controlled read-out.

## 1. Introduction

Due to their central role in inflammatory-based diseases, cytokines are considered as potential biomarkers for cancer, asthma, and several other diseases [1]. The cytokine interleukin-13 (IL-13) plays a central role in respiratory inflammation [2,3] and is a target of new anti-asthmatic drugs [4,5]. Therefore, monitoring of IL-13 concentration could be of high interest within the scope of therapy success. Currently, cytokines are monitored via enzyme-linked immunosorbent assays (ELISA) or flow cytometry. One major drawback of these techniques is the long and extensive workflow [1]. In this respect, the development of a cheap and easy-to-use IL-13 biosensor system would be beneficial.

Especially electrochemical immunosensors could be suitable, as they have the potential to be integrated into a fluidic system and can be automated [6,7,8]. These sensors use antibodies as biological recognition elements and take advantage of the specific antibody–antigen interaction to enable a highly specific and sensitive detection. Since the early development of electrochemical immunoassays in the 1980s [9], this field has grown [10] and is still investigated intensively, as current reviews demonstrate [11,12,13,14]. Main aspects of ongoing research deal with signal amplification strategies, multiplexing approaches, as well as paper-based and fluidic systems [13].

Up to now, research has focused primarily on sensor development rather than of system development. One example of an electrochemical immunosensor system, developed by Schuhmacher et al., comprises a lab-on-chip system for multi-parameter analysis with optical and electrochemical read-out [15]. Further examples are a microfluidic immunosensor array for cytokine and C-reactive protein (CRP) determination [16], and a microfluidic immunosensor platform that allows multiplexed antibiotic detection [17]. Moreover, electrochemical immunosensor systems were used for on-line monitoring of bioreactor products [18] as well as for at-line monitoring of herbicide residues in water [19].

Most immunosensors use enzymes bound to the detection antibodies for signal generation and amplification, but hardly any electrochemical immunosensor or system pays attention to the temperature or pH optimum of the enzyme. Besides enzyme kinetics depending on temperature, temperature influences the rate of the electrode reaction and affects transport processes of involved chemical substances to the electrode surface [20,21]. In the literature, an electrochemical immunosensor with temperature-controlled binding of antigen and antibody has been described [22]. By using heated electrodes, Lou et al. investigated the temperature effect on the lower limit of detection of an electrochemical immunosensor [23], whereas Lau et al. could improve the substrate specificity of an electrochemical enzyme sensor [21,24]. All these aforementioned sensors have in common that they are not integrated into a fluidic system. In addition, fluidic systems with temperature-controlled electrochemical sensors were also reported in literature, for example a hot-wire flow detector with enhanced amperometric response [25] or a thermostat-controlled electrochemical flow cell with heavy metal sensors [26]. However, to the best of our knowledge, up to now no electrochemical immunosensor has been integrated into a thermostat-controlled fluidic system.

Hence, the aim of this work was to develop an electrochemical immunosensor and to integrate it into a thermostat-controlled fluidic system for read-out. Accordingly, two sets of results are reported here. First, an electrochemical immunosensor with an enzyme for read-out was developed to detect IL-13 as a model protein of the cytokine family. Second, a fluidic system with temperature control realized by Peltier elements was built up to enable a temperature-controlled read-out at the temperature optimum of the sensors enzymatic label. For this purpose, the immunosensor was prepared in batch and amperometric read-out was performed in a temperature-controlled fluidic system to evaluate the influence of the temperature on the enzyme activity and thus on the electrochemical sensor response. Under operation at the temperature optimum of the enzyme, an enhanced sensitivity of the electrochemical immunosensor was assumed. Finally, the immunosensor selectivity was evaluated in a complex biological matrix.

## 2. Materials and Methods

### 2.1. The Sensor Principle in Detail

**Figure 1:**
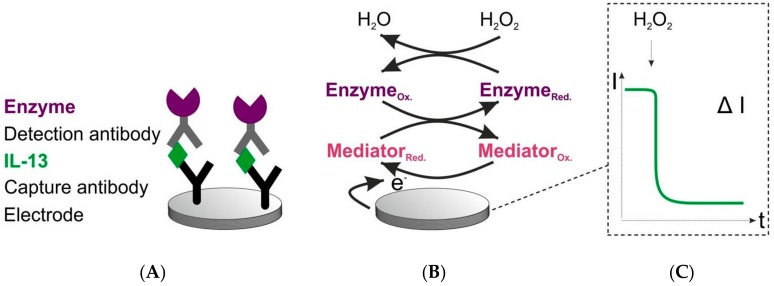
Scheme of sensor set-up and signal generation. (**A**) Sensor set-up. Capture antibodies are covalently immobilized on a single-walled carbon nanotube (SWCNT) working electrode. Detection antibodies are biotinylated. The enzyme (violet) used for read-out is a streptavidin–horseradish peroxidase (HRP) conjugate. (**B**) Signal generation of the sensor. Reaction is initiated by adding hydrogen peroxide. Ox. = oxidized form. Red. = reduced form. Mediator (red.) = hydroquinone, mediator (ox.) = quinone. (**C**) Recorded signal. Change in current over time, when hydrogen peroxide is added.

The sensor architecture and signal generation are depicted in Figure 1. The IL-13 sensor was set up in a sandwich format. The capture antibodies were covalently immobilized on carboxyl-functionalized single-walled carbon nanotube (SWCNT) electrodes. These electrodes were chosen due to the ability of covalent immobilization and in order to facilitate fast electron transfer. In the next steps, the antigen IL-13 and the detection antibody were added. A biotinylated detection antibody was applied to enable a streptavidin-horseradish peroxidase (HRP)-based read-out. For signal generation, the established hydrogen peroxide-hydroquinone-HRP system was used [16,27]. The signal generation was started by adding hydrogen peroxide as a substrate for HRP. Due to the distance between the enzyme and the electrode surface, the mediator hydroquinone was required to support the electron transfer to the electrode. By application of a working potential of 0.0 V (Appendix A), a cathodic current (reduction of the oxidized mediator) over time was measurable.

### 2.2. Chemicals

Disodium hydrogen phosphate, sodium phosphate dibasic, tris-(hydroxymethyl)-aminomethane (TRIS), *N*-ethyl-*N*′-(3-dimethylaminopropyl)-carbodiimide (EDC), bovine serum albumin (BSA), Casein, Tween 20, hydrogen peroxide 30%, Triton X-100, Halt Protease & Phosphatase EDTA-free Inhibitor Cocktail, QuantiPro BCA Assay Kit, and hydroquinone were obtained from Sigma-Aldrich (Munich, Germany). PBS pH 7.2 (Gibco), RPMI-1640 medium, and foetal bovine serum (FBS) were obtained from Thermo Fisher Scientific (Schwerte, Germany). Sodium chloride was obtained from Merck (Darmstadt, Germany). 2-(*N*-Morpholino)-ethanesulfonic acid (MES) was purchased from Amresco (Solon, OH, USA). *N*-Hydroxysulfosuccinimide sodium salt (Sulfo-NHS) was obtained from Alfa Aesar (Heysham Lancashire, UK). Immunoreagents were from the human IL-13 DuoSet ELISA Kit obtained from Bio-Techne (Wiesbaden, Germany). For the immunosensor, a streptavidin–horseradish peroxidase (HRP) conjugate (ABIN964537) from antibodies-online was applied (Aachen, Germany).

### 2.3. Electrochemical System

Screen-printed electrodes (SPE) with carboxyl-functionalized single-walled carbon nanotubes (DRP-110SWCNT) as working electrode (d = 4 mm) were purchased from Dropsens (Llanera, Spain). The counter and reference electrode on the sensor strip are made of carbon and silver, respectively. All potentials are given vs. the reference electrode on the sensor strip. For electrochemical experiments, a PalmSens3 potentiostat/galvanostat with the appropriate Software PSTrace 5.1 was obtained from PalmSens (Houten, The Netherlands).

### 2.4. Performance of Reference ELISA

Before immunosensor preparation, the human IL-13 DuoSet ELISA Kit (Bio-Techne) was used to conduct an ELISA with optical read-out as reference according to the manufacturer’s protocol, which is briefly described as follows. All steps of the procedure were performed at room temperature. Per well, 100 μL of capture antibody were incubated overnight, followed by washing and blocking with BSA solution for 1 h. After washing, 100 μL of IL-13 standard solution was added per well and incubated for 2 h. Subsequently, washing was performed and incubation with 100 μL detection antibody per well for additional 2 h. Once washed, 100 μL of the streptavidin–HRP conjugate was added per well and incubated for 20 min. Then, washing was performed followed by the addition of 100 μL recommended substrate solution, per well, containing 3,3′,5,5′-Tetramethylbenzidine (TMB) and H_2_O_2._ For substrate conversion, 20 min was given, avoiding direct light. Finally, the reaction was stopped by the addition of 50 μL of 2 M sulfuric acid. Read-out was done at 450 nm and at 540 nm with a microplate reader SpectraMax^®^ M3 from Molecular Devices (Sunnyvale, CA, USA). Values at 540 nm were subtracted from values at 450 nm to correct for optical imperfections of the plate.

### 2.5. Immunosensor Preparation

The immunosensor preparation was accomplished in an in-house designed chamber of polyether ether ketone (PEEK), with all steps performed at room temperature. The chamber was blocked before use with 0.1 M PBS pH 7.4 plus 1% (w/v) BSA for 15 min. The 0.1 M PBS solution was composed of disodium hydrogen phosphate, sodium phosphate dibasic, and sodium chloride, modified according to [28]. The immunosensor was set up in a sandwich format according to Figure 1, with the capture and detection antibodies used from the ELISA kit, while the streptavidin-HRP conjugate from antibodies-online (ABIN964537) was employed for read-out. The here-applied concentrations of these immunoreagents were determined in preliminary experiments (data not shown). The capture antibodies were covalently immobilized to the carboxyl-functionalized single-walled carbon nanotubes (SWCNT) on the electrode surface according to the following protocol. The electrodes were washed twice with 100 μL of 25 mM MES buffer pH 5 for 10 min. Carboxylic groups were activated with a mixture of 25 μL of 50 mM EDC and 25 μL of 50 mM Sulfo-NHS for 35 min. Both reagents were freshly prepared in 25 mM MES buffer pH 5 before usage. Then, the electrodes were washed again twice with 100 μL of 25 mM MES buffer pH 5 for 10 min, followed by an incubation step with 50 μL of 36 μg/mL capture antibody in 25 mM MES buffer pH 5 for 1 h. Subsequently, the electrodes were washed twice with 100 μL of 25 mM MES buffer pH 5 for 10 min and residual carboxylic groups were inactivated by washing four times each 15 min with 100 μL of 0.1 M TRIS buffer pH 7.4. Then, the electrodes were washed with 100 μL of 0.1 M PBS pH 7.4 with 0.1% (w/v) BSA and 0.1% (v/v) Tween 20 (storage buffer), compare [29], for 10 min. They were stored in 50 μL of this buffer at 4 °C until use on the next day. The electrodes were stored in the chamber sealed with Parafilm^®^. Before continuing with the immunosensor set-up, a successful immobilization of capture antibodies was verified. Specially, for this purpose, prepared electrodes with capture antibodies were applied. To proof the successful immobilization of capture antibodies, a species-specific antibody labelled with HRP against the capture antibody (ABIN101754 from antibodies-online) was used. Amperometric measurements of the electrodes were performed according to 2.6.

For further immunosensor set-up, electrodes were washed with 100 μL of 0.1 M PBS pH 7.4 to remove residual storage buffer. Then, the electrodes were blocked with 100 μL of a 2% (v/v) Casein in 0.1 M PBS pH 7.4 for 20 min prepared from a 5% (w/v) Casein stock solution, compare [22], to prevent unspecific binding of the antigen to the electrode surface. Next, the electrodes were incubated with 50 μL of defined IL-13 solutions in 0.1 M PBS pH 7.4 plus 1% (w/v) BSA for 1 h. Afterwards, electrodes were washed twice with 100 μL of the storage buffer for 10 min. To complete the sandwich set-up, 50 μL of 5.0 μg/mL of the detection antibody and subsequently 50 μL of 0.5 μg/mL of the streptavidin-HRP conjugate (ABIN964537 from antibodies-online) were added in the next two steps to each sensor. In these steps, dilution to the working concentration, incubation, and washing were done as before for the antigen (see above). To remove residual storage buffer, the electrodes were finally washed with 100 μL of 0.1 M PBS pH 7.4 and were stored at 4 °C until measurement, from a few minutes up to six hours.

### 2.6. Measurement and Characterization of Immunosensors

#### 2.6.1. Measurements in Beaker

Amperometric measurements were conducted in a beaker containing 10 mL of 1 mM hydroquinone in 0.1 M PBS pH 6 under magnetic stirring. The applied working potential was 0.0 V, which was determined as described in the Appendix A. After settling of the background current, the detection was started by adding 100 μL of 50 mM hydrogen peroxide in 0.1 M PBS pH 6 to reach a final concentration of 0.5 mM hydrogen peroxide. The signal was recorded until a steady state was reached, typically within 100 s. For data analysis, the last 100 s of the baseline and the last 100 s of the steady state current were averaged and subtracted from each other. This was repeated for three different immunosensors prepared in the same manner (triplicates), unless otherwise stated. The mean current change and the standard deviation were calculated from these three results. Finally, the mean current changes vs. IL-13 concentration were fitted using the four-parameter logistic model (1) [30]. For calculating the limit of detection (LOD), this equation was solved for *x* and the inserted value for *y* in μA was the sum of the mean of the blank and three times the standard deviation of the blank [31]. Here, the standard deviation of the blank was multiplied by a factor of 3 according to a confidence level of 99%.

Equation of four-parameter logistic model:

(1) $$y=\;\frac{A-D}{1+{\left(\frac{x}{C}\right)}^{B}}+\;D$$

#### 2.6.2. Measurements in Flow Cell

The measurements in the beaker were transferred to a flow cell system with minor modifications: Amperometric measurements in the flow cell were conducted by applying a continuous flow of 1 mM hydroquinone in 0.1 M PBS pH 6 with a flow rate of 0.8 mL/min. The applied potential was 0.0 V. To detect, 100 μL of 1.5 mM hydrogen peroxide in 0.1 M PBS pH 6 were added with a flow rate of 0.4 mL/min to reach a final concentration of 0.5 mM hydrogen peroxide when diluted in the continuous flow. The obtained peak signal was recorded until the baseline was reached again. For data analysis, the last 100 s of the baseline were averaged and subtracted from the peak current. This was repeated for three different immunosensors prepared in the same manner (triplicates), unless otherwise stated. The mean current change and the standard deviation were calculated from these three results. Finally, signals were fitted with the four-parameter logistic model as described above.

### 2.7. Design and Set-Up of Thermostat-Controlled Flow Cell

**Figure 2:**
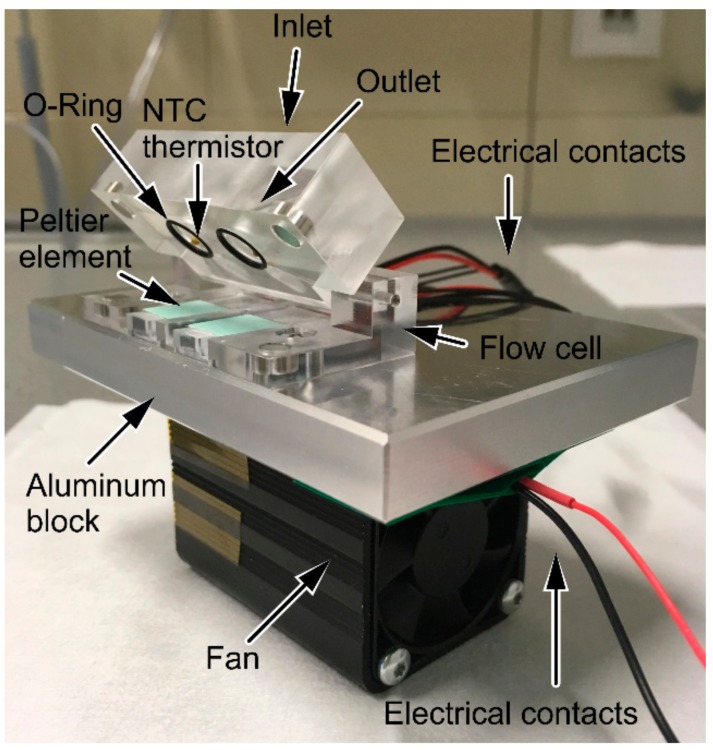
Flow cell for temperature-controlled measurements. Flow cell mounted on aluminum block with fan. Two measurement compartments for dual measurement are available. Temperature control was realized by Peltier elements on the bottom of the cell with thermally conductive pads. In the lid of the cell: inlet, outlet, O-rings, and NTC thermistors. Electrical contacts of the fan and the Peltier elements are shown as well.

The flow cell was designed with the CAD Software Solidworks^®^ 2016 from Dassault Systemes (Vélizy-Villacoublay, France) and was composed of poly-(methyl methacrylate) (PMMA) (Figure 2). To realize temperature control, Peltier elements (CP08,31,06,L1,W4.5) from LairdTech (London, UK) and NTC thermistors (NTCLE305E4103SB) from Vishay (Selb, Germany) were used. Temperature was controlled by applying a TEC controller and the appropriate TEC Service Software from Meerstetter Engineering (Rubingen, Switzerland). The deviation from the set temperature value was ±0.12 °C in the range of 12 °C to 37 °C. The measurement compartment (volume 80 μL) was sealed with an O-ring (d = 7 mm). The flow cell was mounted on an aluminum block; for better heat dissipation, a fan was attached to the aluminum block. To set up a flow injection analysis (FIA) system, the flow cell was connected to a neMESYS syringe pump system from Cetoni (Korbussen, Germany) and the appropriate neMESYS UserInterface Software was used.

### 2.8. Selectivity of the IL-13 Immunosensor

To evaluate the selectivity of the IL-13 immunosensor, cell lysate of human lung cancer cell line NCI-H1975 was prepared. Cells were cultured in RPMI-1640 medium with 10% (v/v) FBS and kindly provided by a colleague. For lysate preparation the cells were rinsed two times with PBS pH 7.2 (Gibco by Life Technologies) and lysed by adding 1 mL of lysis buffer (pH 7.4, 150 mM NaCl, 50 mM Tris, 1% (v/v) Triton X-100, 1x Halt Protease & Phosphatase EDTA-free Inhibitor Cocktail), compare [32], to each T75 cell culture flask and by incubating at 4 °C for 60 min under shaking. Then, cell fragments were removed by centrifugation at 15,000× *g* and 4 °C for 30 min. Total protein concentration was determined by applying the QuantiPro BCA Assay Kit (Sigma Aldrich, Munich, Germany). Lysates were stored until use at −80 °C. For selectivity determination, IL-13 immunosensors were prepared according to Section 2.5. Therefore, the lysate was normalized to a total protein amount of 5 μg and spiked with defined IL-13 concentrations. For comparison 0.1 M PBS pH 7.4 plus 1% (w/v) BSA with the same IL-13 concentrations was analyzed. Amperometric measurements were conducted in the flow cell, without temperature control, according to Section 2.6.2.

## 3. Results and Discussion

### 3.1. Performance of Reference ELISA

**Figure 3:**
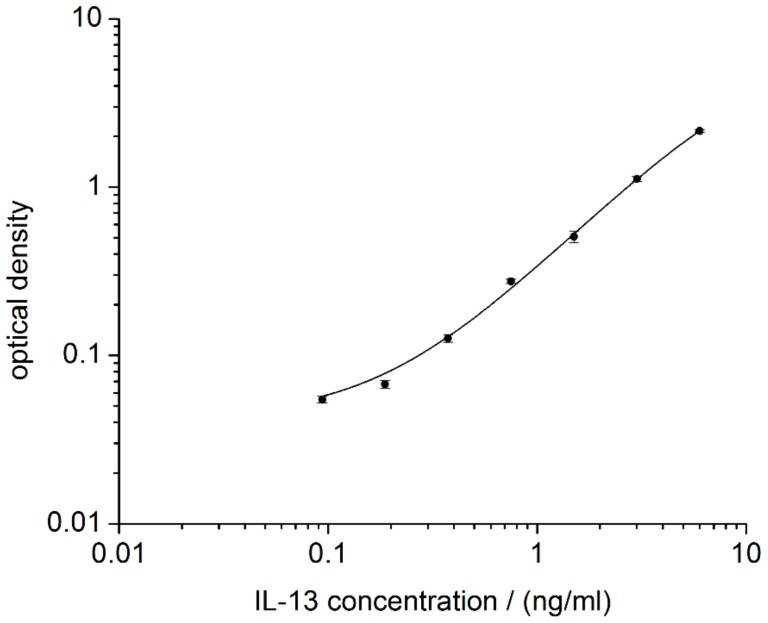
IL-13 ELISA as reference. Logarithmic representation of optical density vs. IL-13 concentration. For each concentration, duplicates were prepared according to the manufacture’s protocol. Read-out was performed at 450 nm and at 540 nm. Values at 540 nm were subtracted from values at 450 nm to correct for optical imperfections of the plate.

Prior to the development of an IL-13 immunosensor, the immunoreagents form the ELISA kit were checked by performing an ELISA according to the manufacturer’s protocol (2.4). The calibration curve obtained is in line with the manufacturer’s specifications and a LOD of 99 pg/mL was calculated (Figure 3). The assay spanned a dynamic range from 99 pg/mL of IL-13 to 6000 pg/mL of IL-13. Compared with IL-13 concentrations in blood for healthy (20 pg/mL) and for asthmatic patients (120 pg/mL) [33,34], only concentrations for asthmatic patients are within the measurable range of the ELISA. Nevertheless, it was proven that the immunoreagents are suited for IL-13 determination and that they could be the basis for the development of a respective electrochemical immunosensor.

### 3.2. Sensor Characterization in Beaker

**Figure 4:**
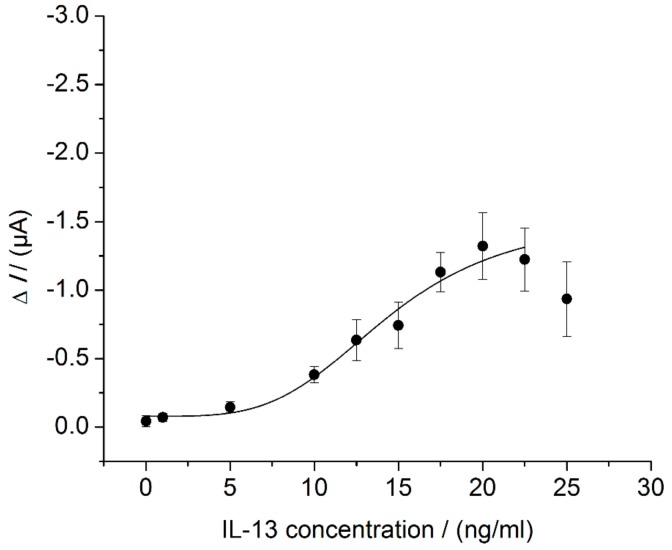
Characterization of the electrochemical IL-13 immunosensor in the beaker. Three different immunosensors prepared in the same manner (triplicates) for each concentration. Amperometric measurements performed at 0.0 V. Measurement in 10 mL of 1 mM hydroquinone in 0.1 M PBS pH 6 with 100 μL of 50 mM hydrogen peroxide in 0.1 M PBS pH 6 added to start read-out. Measurements at 25 ng/mL of IL-13 are not included in curve fitting.

The electrochemical IL-13 immunosensor was set up as described above (Section 2.1 and Section 2.5). For sensor characterization, different IL-13 concentrations were measured according to Section 2.6.1. A sigmoid relationship between the measured current and the IL-13 concentration was found (Figure 4). The maximum sensor signal is reached at 20 ng/mL of IL-13 and decreases for higher concentrations of IL-13 up to 25 ng/mL. A decreasing signal with increasing antigen concentration is well known from one-stage immunoassays, where the antigen and detection antibody are added simultaneously [35]. This so-called Hook-Effect describes the occurrence of false-negative signals in immunoassays caused by an excess of antigen. This effect may also occur in assays where antigen and detection antibody are added sequentially. Here, this phenomenon could be due to incomplete washing after incubation with the antigen [36]. This may be an explanation of the signal decrease at high IL-13 concentrations described in this work. Since the lower concentration range is more relevant for the mentioned application of the sensor, the signal decrease at higher IL-13 concentrations is not crucial.

For the IL-13 immunosensor, a LOD of 7.0 ng/mL of IL-13 was calculated. Hence, the here-developed immunosensor is 70-fold less sensitive compared to the conventionally performed ELISA (3.1). Moreover, the sensor showed a small dynamic range from 7.0 ng/mL to 20 ng/mL of IL-13, which is at the upper end of the dynamic range of the ELISA (6 ng/mL, see Figure 3). Despite using the same antibodies for the ELISA and the sensor, the two methods show various differences, e.g., the surfaces where the antibodies are immobilized (polystyrene vs. SWCNT) and the immobilization techniques (adsorptive vs. covalent), which could be responsible for the observed differences in method performance. In both cases, a blocking step was performed either with BSA solution or with casein solution. The BSA solution was prescribed in the manufacturer’s protocol of the ELISA. The casein solution was chosen for sensor preparation, because it was recommended in literature for an immunosensor based on SWCNT electrodes [22]. Additionally, there is a difference in the applied streptavidin–HRP conjugates. For the ELISA, the streptavidin–HRP conjugate from the kit was used. For this conjugate, information on the concentration was not given and hence a streptavidin–HRP conjugate with specified concentration (ABIN964537 from antibodies-online) was utilized for the sensor. Besides this, the ELISA and sensor differ in the enzymatic substrates (TMB and H_2_O_2_ vs. hydroquinone and H_2_O_2_) and the read-out methods (optical vs. electrochemical). Hydroquinone was chosen over TMB for the sensor to avoid passivation and poising caused by oxidized TMB [37]. During optical read-out, the resulting optically active product is directly measurable. During electrochemical read-out, the product is not measured, but rather the resulting electron flow from the electrode to the enzymatically generated oxidized mediator (quinone). Regarding the main sensor components, electrodes, immobilization techniques, enzymatic labels, and substrates, as well as electrochemical read-out methods and instruments, Ricci et al. give advice on the challenging task of electrochemical immunosensor development [37]. Furthermore, they point out that a simple adaptation of an ELISA to an electrochemical immunosensor rarely leads to improvements concerning sensitivity, limit of detection, time to result and costs.

**Figure A3:**
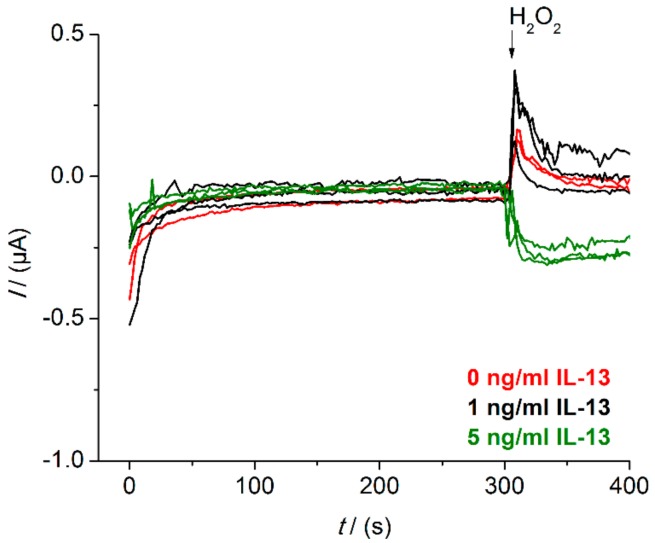
Raw data signals of IL-13 immunosensor measured in the beaker. Amperometric measurements performed at 0.0 V. Triplicates of sensors with 5.0 ng/mL of IL-13 (**green**) and with 1.0 ng/mL of IL-13 (**black**) prepared. Duplicates of sensor with 0.0 ng/mL of IL-13 (**red**) prepared. Measurements in 10 mL of 1 mM hydroquinone in 0.1 M PBS pH 6.0 after addition of 100 μL of 50 mM hydrogen peroxide in 0.1 M PBS pH 6.0.

Compared to other electrochemical immunosensors, the developed IL-13 sensor is also less sensitive. One reason could be missing signal amplification. Other groups using poly-HRP conjugates for cytokine detection [38] or advanced detection antibody conjugates comprising magnetite (Fe_3_O_4_) nanoparticles on graphene oxide sheets for cancer biomarker detection [39] reach detection limits of pg/mL or even fg/mL. Another reason could be a relatively high and noisy background signal (Appendix B, Figure A3), which makes it difficult to discriminate between small IL-13 concentrations and the background.

In summary, the here-developed IL-13 immunosensor is less sensitive compared to ELISA and to other electrochemical immunosensors. With the aim to enhance the sensitivity of the sensor, a temperature-controlled read-out was introduced. For this purpose, a fluidic system with temperature control was developed. Further, the fluidic system is suitable to minimize manual interaction. Results are described below.

### 3.3. Development of a Fluidic System with Temperature Control

#### 3.3.1. Determination of Flow Parameters

**Figure 5:**
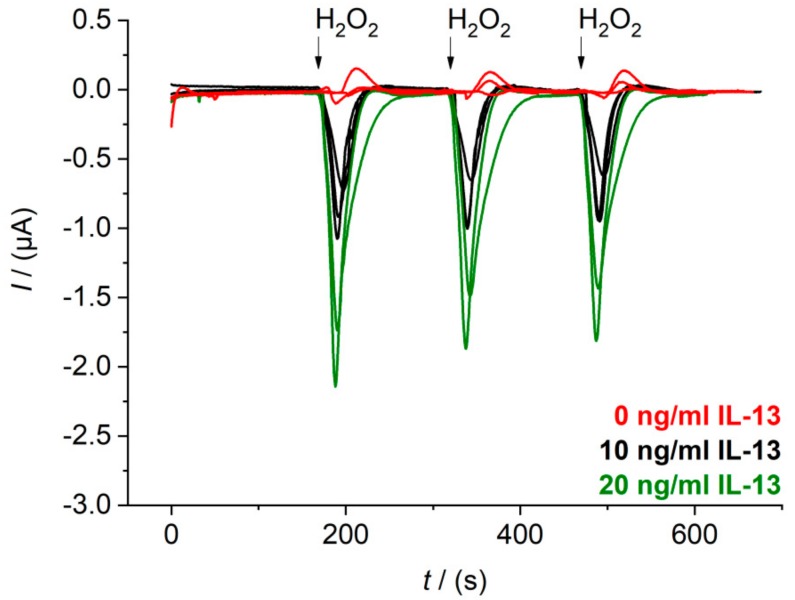
Raw data signals of the electrochemical IL-13 immunosensor measured in the flow cell. Amperometric measurements performed at 0.0 V. Duplicates of sensor with 20 ng/mL of IL-13 (**green**), triplicates of sensor with 10 ng/mL of IL-13 (**black**) and with 0.0 ng/mL of IL-13 (**red**) prepared. Each sensor measured three times in series. Continuous flow of 1 mM hydroquinone in 0.1 M PBS pH 6.0 with 0.8 mL/min, injection flow of 1.5 mM hydrogen peroxide in 0.1 M PBS pH 6 with 0.4 mL/min and injection volume of 100 μL.

**Figure A4:**
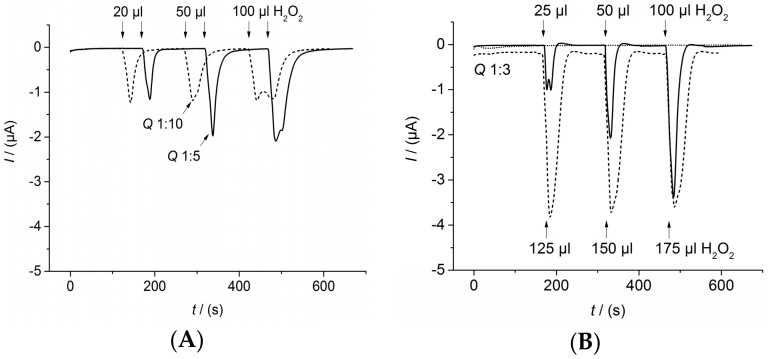
Determination of flow parameters. Amperometric measurements performed at 0.0 V: (**A**) total flow 1 mL/min. For *Q* 1:10 (dashed line), continuous flow of 1 mM hydroquinone in 0.1 M PBS pH 6.0 with 0.9 mL/min, injection flow of 1.5 mM hydrogen peroxide in 0.1 M PBS pH 6.0 with 0.1 mL/min. For *Q* 1:5 (solid line), continuous flow of 1 mM hydroquinone in 0.1 M PBS pH 6.0 with 0.8 mL/min, injection flow of 1.5 mM hydrogen peroxide in 0.1 M PBS pH 6.0 with 0.2 mL/min. Injection volume 20, 50, and 100 μL; (**B**) total flow 1.2 mL/min. For *Q* 1:3 (dashed and solid line), continuous flow of 1 mM hydroquinone in 0.1 M PBS pH 6.0 with 0.8 mL/min, injection flow of 1.5 mM hydrogen peroxide in 0.1 M PBS pH 6.0 with 0.4 mL/min. Injection volume 25, 50, 100, 125, 150, and 175 μL. For *Q* 1:3 (dotted line), continuous flow of 1 mM hydroquinone in 0.1 M PBS pH 6.0 with 0.8 mL/min, injection flow of 1 mM hydroquinone in 0.1 M PBS pH 6.0 with 0.4 mL/min and injection volume 25, 50, and 100 μL.

The flow cell with temperature control was designed and was set up as described in Section 2.7. In a first step, flow rates and injection volume were optimized to get defined peak signals during sensor measurement (Appendix C). A continuous flow of 0.8 mL/min of 1 mM hydroquinone in 0.1 M PBS pH 6.0 was chosen as well as an injection volume of 100 μL of 1.5 mM hydrogen peroxide in 0.1 M PBS pH 6.0. The hydrogen peroxide solution was injected with a flow rate of 0.4 mL/min to reach a final concentration of 0.5 mM, when diluted in the continuous flow. This final hydrogen peroxide concentration is similar to the final concentration in the beaker measurement. Raw data signals of the immunosensor with three different IL-13 concentrations obtained with the chosen flow parameters are depicted in Figure 5. Sensors were prepared in triplicates (for 0.0 ng/mL and 10 ng/mL of IL-13) or at least in duplicates (for 20 ng/mL of IL-13). Furthermore, each sensor was measured three times in series. For sensors with 0.0 ng/mL of IL-13, small oxidative current peaks were observed. Injection of background buffer without H_2_O_2_ proved that these peaks are not caused by the injection event itself (Figure A4B). They may be ascribed to changes of the redox equilibrium of electrode surface groups by the addition of the strong oxidizing agent H_2_O_2_. For the sensors with 10 ng/mL of IL-13 and 20 ng/mL of IL-13 defined reduction peaks were obtained with a slight signal decrease for the three measurements in series. Possibly, some HRP molecules were inactivated through suicide inhibition by hydrogen peroxide, which is characteristic for peroxidases [40].

**Figure 6:**
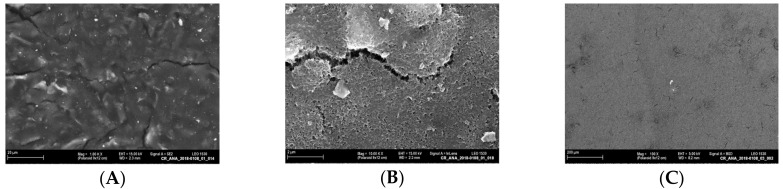
SEM images of SWCNT electrodes recorded at different excitation voltages and with different detectors: (**A**) resolution 1000×, excitation voltage 15 kV, SE detector; (**B**) zoomed in section of image (**A**), resolution 10,000×, excitation voltage 15 kV, SE InLens detector; (**C**) other electrode, resolution 100×, excitation voltage 5 kV, BSD detector, residues of organic solvents are visible as a dark shade.

Furthermore, the signals of the replicates for each IL-13 concentration varied remarkably. This could be caused by variations during sensor preparation or by quality problems of the screen-printed SWCNT electrodes. Printing of highly viscous inks such as SWCNT ink is challenging and leads to quality issues [41,42]. Moreover, the quality of screen-printed electrodes is generally lower, when compared to other electrode fabrication techniques [43]. A non-homogeneous electrode surface was confirmed via SEM (Figure 6A,B), and residues of organic solvents were identified (Figure 6C).

In summary, flow parameters were optimized and manual influence was reduced using the flow cell set-up.

#### 3.3.2. Application of the Fluidic System for Sensor Measurements

**Figure 7:**
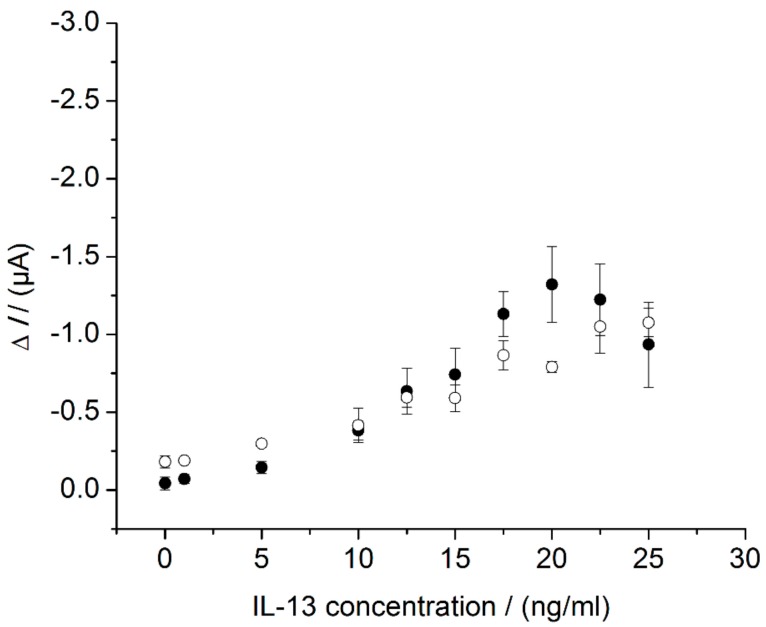
Comparison of immunosensor characteristics in beaker and in flow cell. Three different immunosensors prepared in the same manner (triplicates) for each concentration. Amperometric measurements performed at 0.0 V. Measurements in beaker (black circles) in 10 mL of 1 mM hydroquinone in 0.1 M PBS pH 6, after addition of 100 μL of 50 mM hydrogen peroxide in 0.1 M PBS pH 6. Measurements in flow cell (white circles) with continuous flow of 1 mM hydroquinone in 0.1 M PBS pH 6 with 0.8 mL/min, injection flow of 1.5 mM hydrogen peroxide in 0.1 M PBS pH 6 with 0.4 mL/min and injection.

After suitable flow parameters were determined, sensors were measured in the flow cell according to Section 2.6.2. The results were compared with the sensor measurements in the beaker (Figure 7). The overall sensor response of the peak amplitudes measured in the flow cell (white circles) and the steady-state currents measured in the beaker (back circles) were similar: In the flow cell, a LOD of 5.8 ng/mL was calculated; in the beaker, a LOD of 7.0 ng/mL was found out (Section 3.2). Besides similar LOD, the slope of the calibration curve differed slightly for the two systems. An almost linear relationship between the current and the IL-13 concentration was found for the flow cell measurement, without a signal decrease at high IL-13 concentrations, whereas a more sigmoid relationship between the current and the IL-13 concentration, with a signal decrease at high IL-13 concentrations, was observed in the beaker. Aside from a possible Hook-Effect (compare Section 3.2), there could be another explanation for the decreasing signal. In the case of high IL-13 concentrations, the sensor surface is densely covered with detection antibodies and HRP molecules. As a consequence, hydroquinone molecules concentration near the sensors surface could be depleted, which is relevant for signal generation and read-out. Hence, stirring of the hydroquinone solution in the beaker could be insufficient to cope with the depletion. When applying the flow cell, the superimposed flow of hydroquinone solution above the sensor surface might reduce the depletion zone. This positive effect of a superimposed flow, originally described for binding reactions [44,45], might also be applicable to (electro-) chemical reactions.

At this stage, the results obtained with the beaker and with the flow cell behave rather similar and each system can be used for immunosensor measurements. However, the flow cell offers a higher potential for future work, as manual interactions are minimized and automated measuring is possible. In the next step, the sensor system was applied for a temperature-controlled read-out to assess the influence of temperature on the amperometric signal.

### 3.4. Sensor Characteristics in Thermostat-Controlled Flow Cell

**Figure 8:**
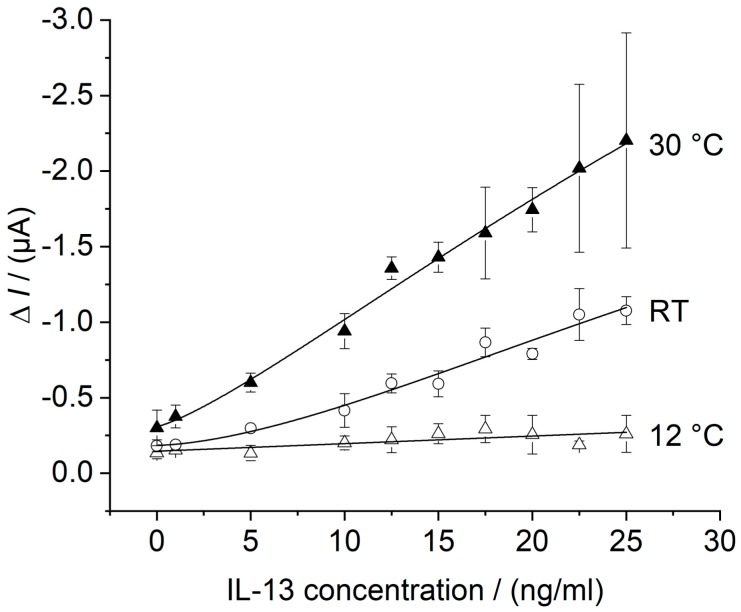
Calibration curves of the IL-13 immunosensor in thermostat-controlled flow cell. Three different immunosensors prepared in the same manner (triplicates) for each concentration. Amperometric measurements performed at 0.0 V. Measurements in flow cell with continuous flow of 1 mM hydroquinone in 0.1 M PBS pH 6 with 0.8 mL/min, injection flow of 1.5 mM hydrogen peroxide in 0.1 M PBS pH 6 with 0.4 mL/min and injection volume of 100 μL. Measurements conducted at 12 °C (white triangles) and at 30 °C (black triangles). Measurements without temperature control (i.e., at RT = room temperature) from Figure 7 (white circles) are shown for comparison. Calibration curve at 12 °C was fitted with a linear model, whereas the calibration curves at RT and 30 °C were fitted with the 4-parameter logistic model, as described in Section 2.6.

Using the flow cell with temperature control the calibration curve of the in batch prepared IL-13 immunosensor was recorded at the temperature optimum of HRP at 30 °C [46,47] and at 12 °C as a reference for lower performance. The temperature optimum was determined in preliminary experiments (please see Appendix D). Measurements were conducted according to Section 2.6.2 and results are depicted in Figure 8. For comparison, the calibration curve without temperature control, already shown in Figure 7, is depicted as well.

For the calibration curve at 30 °C, an almost linear relationship between the measured current and the IL-13 concentration was found and a LOD of 5.4 ng/mL of IL-13 was calculated. Furthermore, there is a small offset of about 0.1 μA at 0.0 ng/mL of IL-13 when compared to the curves at 12 °C and without temperature control. The curve at 30 °C showed the highest signal increase compared to the lower temperatures. By considering the standard deviations at the upper concentration range, concentrations higher 12.5 ng/mL of IL-13 could not be distinguished reliably. For the calibration curve at 12 °C, a linear relationship between the measured current and the IL-13 concentration was observed for the entire concentration range with a slope of 0.005 μA/(ng/mL).

The signal increase with higher temperature reflects the higher activity of the enzymatic label HRP. Higher signals could be advantageous to improve the detection limit. Besides enhanced enzyme activity, temperature increase supports mass transfer and electron transfer, which may contribute to the observed offset. Further, temperature control in general may be convenient to reduce variations of the sensor response as described in literature [19]. Unsatisfying is an almost unchanged LOD of 5.4 ng/mL for the calibration curve at 30 °C compared to a LOD of 5.8 ng/mL without temperature control. Hence, a temperature increase led to a higher sensitivity (slope); however, the detection limit was not improved. The sensor system has to be further optimized in terms of background signal, noise reduction and signal amplification. For the latter, Sanchez-Tirado et al. used for example a streptavidin–poly-HRP conjugate for read-out [38]. This offers several catalytically active HRP molecules per marked antibody instead of one. Concerning background signal and noise, electrochemical issues should be further investigated such as electrode material, selected mediator, and applied potential. This would facilitate the discrimination of small IL-13 concentrations from the background and would lead to an improved LOD. Despite of improvements to be made, the developed immunosensor was applied for IL-13 detection in a complex biological matrix to determine the sensors selectivity.

### 3.5. Selectivity of the IL-13 Immunosensor

**Figure 9:**
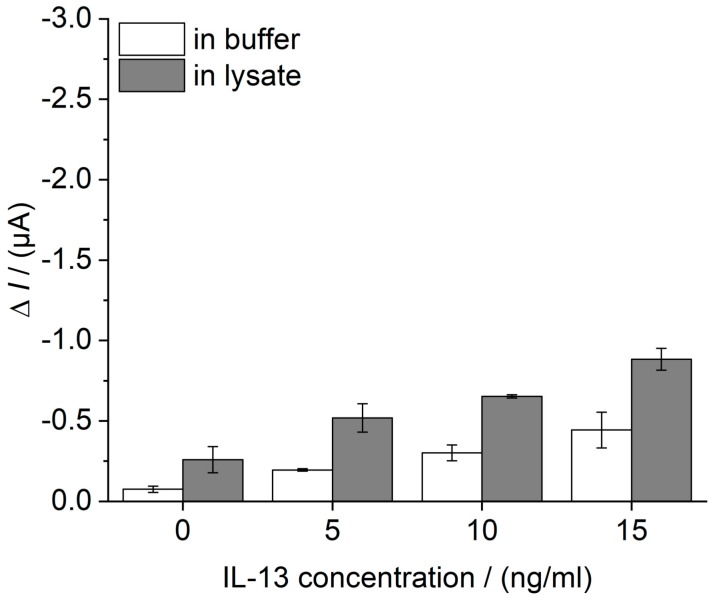
Evaluation of the selectivity of the IL-13 immunosensor. Cell lysate of human lung cancer cell line NCI-H1975, normalized to a total protein amount of 5 μg and spiked with defined IL-13 concentrations (grey). For comparison, 0.1 M PBS pH 7.4 plus 1% (w/v) BSA was spiked with equal IL-13 concentrations (white). Immunosensors were prepared in triplicates. Amperometric measurements performed at 0.0 V and without temperature control, i.e., at room temperature. Measurements in flow cell with continuous flow of 1 mM hydroquinone in 0.1 M PBS pH 6 with 0.8 mL/min, injection flow of 1.5 mM hydrogen peroxide in 0.1 M PBS pH 6 with 0.4 mL/min and injection volume of 100 μL.

In a first selectivity study, the ability of the developed immunosensor to detect IL-13 concentrations in a complex biological matrix was assessed. Therefore, cell lysate of human lung cancer cell line NCI-H1975, spiked with defined IL-13 concentrations (0–15 ng/mL) was analyzed. For comparison, buffer containing equal IL-13 concentrations was tested. Lysate and immunosensor preparation are described in Section 2.8. As a result, buffer and lysate showed an IL-13 concentration-depended sensor signal (Figure 9). Moreover, a small matrix effect was identified for the lysate.

## 4. Conclusions

The here-presented approach addresses a temperature-optimized read-out of an amperometric IL-13 immunosensor in a fluidic system for the first time. With a temperature optimum at 30 °C for read-out, a clear improvement of sensor performance by temperature control could be demonstrated, although the amperometric immunosensor system, with a LOD of 5.4 ng/mL of IL-13 at 30 °C, showed to be 55-fold less sensitive compared to the ELISA. This may be due to differences in the two systems as described. Hence, the IL-13 sensor system could be improved with respect to background signal, noise reduction, and signal amplification. Additionally, the quality of the used SPE should be investigated and the sensor preparation procedure needs to be improved in future work. To apply the sensor in clinics, further selectivity studies as well as clinical studies have to be performed. Although further improvements are to be done, the sensor system is a first step towards an automated immunosensor platform.

## Figures and Tables

**Figure A1:**
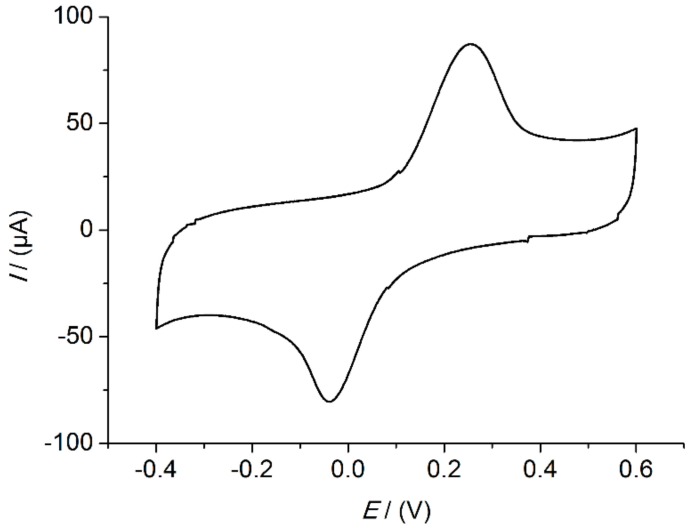
Cyclic voltammogram of SWCNT electrode with built-up immunosensor. Immunosensor on SWCNT electrode with 10 ng/mL of IL-13. Solution: 1 mM hydroquinone in 0.1 M PBS pH 6.0. Scan rate: 0.1 V/s.

**Figure A2:**
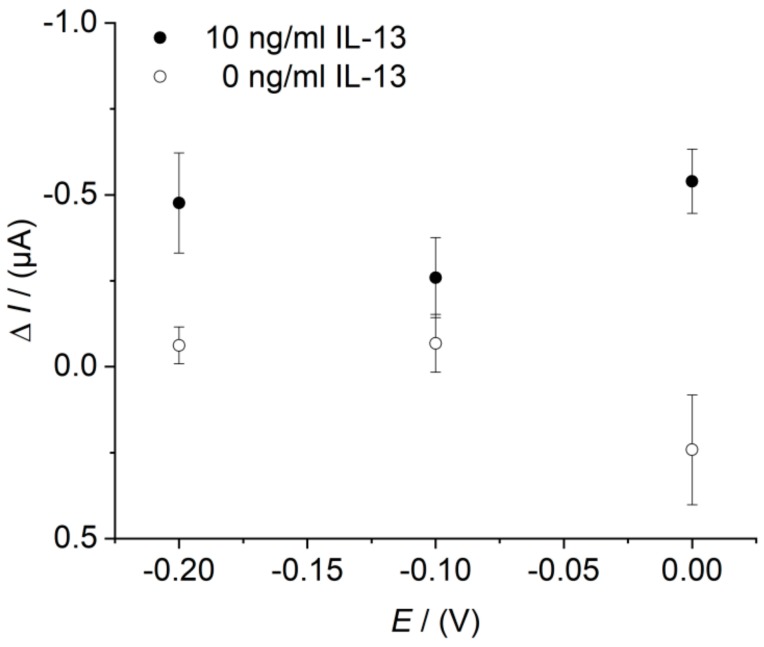
Effect of the working potential on the immunosensor response determined in the beaker. Three different immunosensors prepared in the same manner (triplicates) for each data point. Amperometric measurements performed with 10 ng/mL of IL-13 (black circles) and with 0.0 ng/mL of IL-13 (white circles). Measurements in 10 mL of 1 mM hydroquinone in 0.1 M PBS pH 6.0 after addition of 100 μL of 50 mM hydrogen peroxide in 0.1 M PBS pH 6.0. Potentials of 0.0 V, −0.1 V, and −0.2 V were applied.

**Figure A5:**
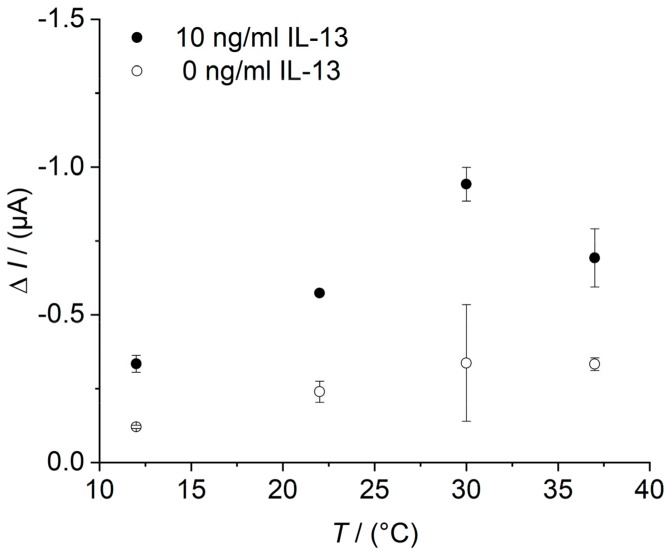
Determination of temperature optimum of the IL-13 immunosensor in the flow cell. Amperometric measurements performed at 0.0 V. Sensor with 10 ng/mL of IL-13 (black circles) and sensor with 0.0 ng/mL of IL-13 (white circles), each measured two times in series at the same temperature. Temperature of 12 °C, 22 °C, 30 °C, and 37 °C applied. Measurements in flow cell with continuous flow of 1 mM hydroquinone in 0.1 M PBS pH 6 with 0.8 mL/min, injection flow of 1.5 mM hydrogen peroxide in 0.1 M PBS pH 6 with 0.4 mL/min, and injection volume of 100 μL.

## Appendix A. Determination of the Working Potential

The applied working potential of 0.0 V was selected based on cyclic voltammetry (CV) measurements, exemplarily depicted in Figure A1. This result was further confirmed in amperometric measurements at various fixed potentials (Figure A2). The sensors were prepared in triplicates as described in Section 2.5. Each sensor was measured at 0.0 V, −0.1 V, and −0.2 V successively. A potential of −0.2 V is often used by other groups [16,27]. In our measurements, an applied potential of 0.0 V showed the highest signal change combined with a low background.

## Appendix B. Raw Data Signals of Measurements in Beaker

The exemplary depiction of raw data signals of IL-13 sensors measured in the beaker shows relatively high and noisy background signals (Figure A3). Therefore, small IL-13 concentrations are hardly distinguishable from the background.

## Appendix C. Determination of the Flow Parameters

For the determination of flow rates and suitable injection volumes, a simpler sensor set-up was used. The capture antibodies were immobilized according to Section 2.5. Instead of the complete immunosensor set-up, only a species-specific antibody labelled with HRP against the capture antibody was added (ABIN101754 from antibodies-online). Different flow rates for the continuous flow of 1 mM hydroquinone in 0.1 M PBS pH 6.0 as well as for the injection flow of 1.5 mM hydrogen peroxide in 0.1 M PBS pH 6.0 were investigated (Figure A4). The dilution factor associated with the flow rate ratio is denoted as *Q*. For example, a ratio of *Q* 1:3 means that one part of the total flow during injection consists of the hydrogen peroxide injection solution and two parts consist of the continuous hydroquinone solution. Furthermore, different injections volumes were tested. The optimal injection volume is given for a high peak signal and minimized tailing. Further, the influence of the injection event itself on the sensor signal was investigated with the same solution for continuous flow and injection but without H_2_O_2_. In this case, no peak was observed during injection (Figure A4B, dotted line). Altogether optimal performance was determined for *Q* 1:3 and an injection volume of 100 μL.

## Appendix D. Determination of the Temperature Optimum of the IL-13 Immunosensor

To determine the temperature optimum of the IL-13 immunosensor read-out, the temperature of the flow cell was controlled in the range of 12 °C to 37 °C and a sensor with a concentration of 10 ng/mL IL-13 has been compared to the reference of 0.0 ng/mL IL-13. For sensor preparation, please see Section 2.5 and for amperometric measurements, see Section 2.6.2. At each temperature, the sensor response was recorded twice. The current signal of the sensor with IL-13 increased until 30 °C and then decreased (Figure A5). The current signal of the sensor without IL-13 showed the same trend but with a less pronounced signal increase. The highest current signal at 30 °C corresponds to the temperature optimum of the IL-13 immunosensor or rather to the temperature optimum of HRP [46,47]. The signal decrease at temperatures higher than 30 °C is presumably related to changes in the tertiary structure of HRP, which accords with circular dichroism studies by Chattopadhyay and Mazumdar [48].
